# Predicting *In vitro* Culture Medium Macro-Nutrients Composition for Pear Rootstocks Using Regression Analysis and Neural Network Models

**DOI:** 10.3389/fpls.2016.00274

**Published:** 2016-03-29

**Authors:** S. Jamshidi, A. Yadollahi, H. Ahmadi, M. M. Arab, M. Eftekhari

**Affiliations:** ^1^Department of Horticulture, Faculty of Agriculture, Tarbiat Modares UniversityTehran, Iran; ^2^Department of Poultry Science, Faculty of Agriculture, Tarbiat Modares UniversityTehran, Iran

**Keywords:** neural network model, *in vitro* culture medium, macro nutrients, regression analysis, optimized medium

## Abstract

Two modeling techniques [artificial neural network-genetic algorithm (ANN-GA) and stepwise regression analysis] were used to predict the effect of medium macro-nutrients on *in vitro* performance of pear rootstocks (OHF and Pyrodwarf). The ANN-GA described associations between investigating eight macronutrients (NO${}_{3}^{-}$, NH${}_{4}^{+}$, Ca^2+^, K^+^, Mg^2+^, PO${}_{4}^{2-}$, SO${}_{4}^{2-}$, and Cl^−^) and explant growth parameters [proliferation rate (PR), shoot length (SL), shoot tip necrosis (STN), chlorosis (Chl), and vitrification (Vitri)]. ANN-GA revealed a substantially higher accuracy of prediction than for regression models. According to the ANN-GA results, among the input variables concentrations (mM), NH${}_{4}^{+}$ (301.7), and NO${}_{3}^{-}$, NH${}_{4}^{+}$ (64), SO${}_{4}^{2-}$ (54.1), K^+^ (40.4), and NO${}_{3}^{-}$ (35.1) in OHF and Ca^2+^ (23.7), NH${}_{4}^{+}$ (10.7), NO${}_{3}^{-}$ (9.1), NH${}_{4}^{+}$ (317.6), and NH${}_{4}^{+}$ (79.6) in Pyrodwarf had the highest values of VSR in data set, respectively, for PR, SL, STN, Chl, and Vitri. The ANN-GA showed that media containing (mM) 62.5 NO${}_{3}^{-}$, 5.7 NH${}_{4}^{+}$, 2.7 Ca^2+^, 31.5 K^+^, 3.3 Mg^2+^, 2.6 PO${}_{4}^{2-}$, 5.6 SO${}_{4}^{2-}$, and 3.5 Cl^−^ could lead to optimal PR for OHF and optimal PR for Pyrodwarf may be obtained with media containing 25.6 NO${}_{3}^{-}$, 13.1 NH${}_{4}^{+}$, 5.5 Ca^2+^, 35.7 K^+^, 1.5 Mg^2+^, 2.1 PO${}_{4}^{2-}$, 3.6 SO${}_{4}^{2-}$, and 3 Cl^−^.

## Introduction

From the inception of plant tissue culture (leaf mesophyll and hair cells) by Austrian botanist Gottlieb Haberlandt (1902) in nutritive media, numerous researches have been done on the optimization of various culture media to provide explants favorite propagation conditions.

Study on the relationship between media nutrients and explant proliferation may result to design a more effective medium. Hence, statistical and mathematical tools such as linear regression, logistic regression, and mixed models are used. Artificial neural network (ANN) is an operative substitute used for trustworthy assessments of the biological systems. Neural network technology estimates different complex mathematical functions to process and infer various sets of irregular data. This technology simulates the structure of the human neuron network as it includes information processing and decision making abilities. They can recognize and model complex non-linear relationships between the input and output of a biological process owing to their high learning aptitude, (Hashimota, 1997; Nazmul Karim et al., 1997; Patnaik, 1999). There are three kinds of layers in ANN including input layer, one or more hidden layers, and output layer. The neurons are linked together with different connection strength. The connections are entitled synaptic weights, in which, all data on the network is encoded as those weights are actually the numbers that regulate the strength of the impulses coming to the neurons. The most critical characteristic of ANN, which specifies that computational method, is the process of training. Training of the networks is recognized with the variations of the values for all synaptic weights using a specific algorithm (Haykin, 1999). The most well-known learning algorithm is back-propagation procedure. In which, the error made because of inconsistencies between the system output (observed) and the expected outcome is propagated back to simplify readjustments of the weights allocated to the connections up to the network attains an appropriate generality (Dayhoff and DeLeo, 2001). Currently, the ANN has been used in many fields to model and anticipate the performances of systems, based on certain input–output data. Whereas, neural networks have presented considerable advances in the field of computer-generated adjustment of bioprocesses, their applications to complex *in vitro* plant culture systems are relatively new and limited only to a small number of cases (Prasad and Dutta Gupta, 2006). It is essential to optimize the factors influencing plant tissue culture systems. Acquired optimized models represent appropriate resolutions when trained with a set of special constraints. These constraints weights are saved in the connections so that feeding with independent variables causes the network predicts the relationship of variables which results in optimum solution (Prasad and Dutta Gupta, 2006).

In this study, we demonstrated a use of statistical method of experimental design, Taguchi method, in inspecting the optimum macronutrientś concentrations for explant proliferation and ANN to evaluate the effect and importance of several essential mineral elements' concentration on explant proliferation to: (1) develop an ANN-based model to answer questions relating to the appropriate macronutrient concentrations for achieving the most suitable results for the parameters studied, (2) apply the developed ANN-model to evaluate the relative importance of the studied ions, on proliferation, and (3) optimize ANN-model to find the optimum values of input variable for maximizing PR. This could be used to carry out proper culture media optimization studies, in this case, for these *Pyrus* rootstocks' micro-propagation.

**Figure 1:**
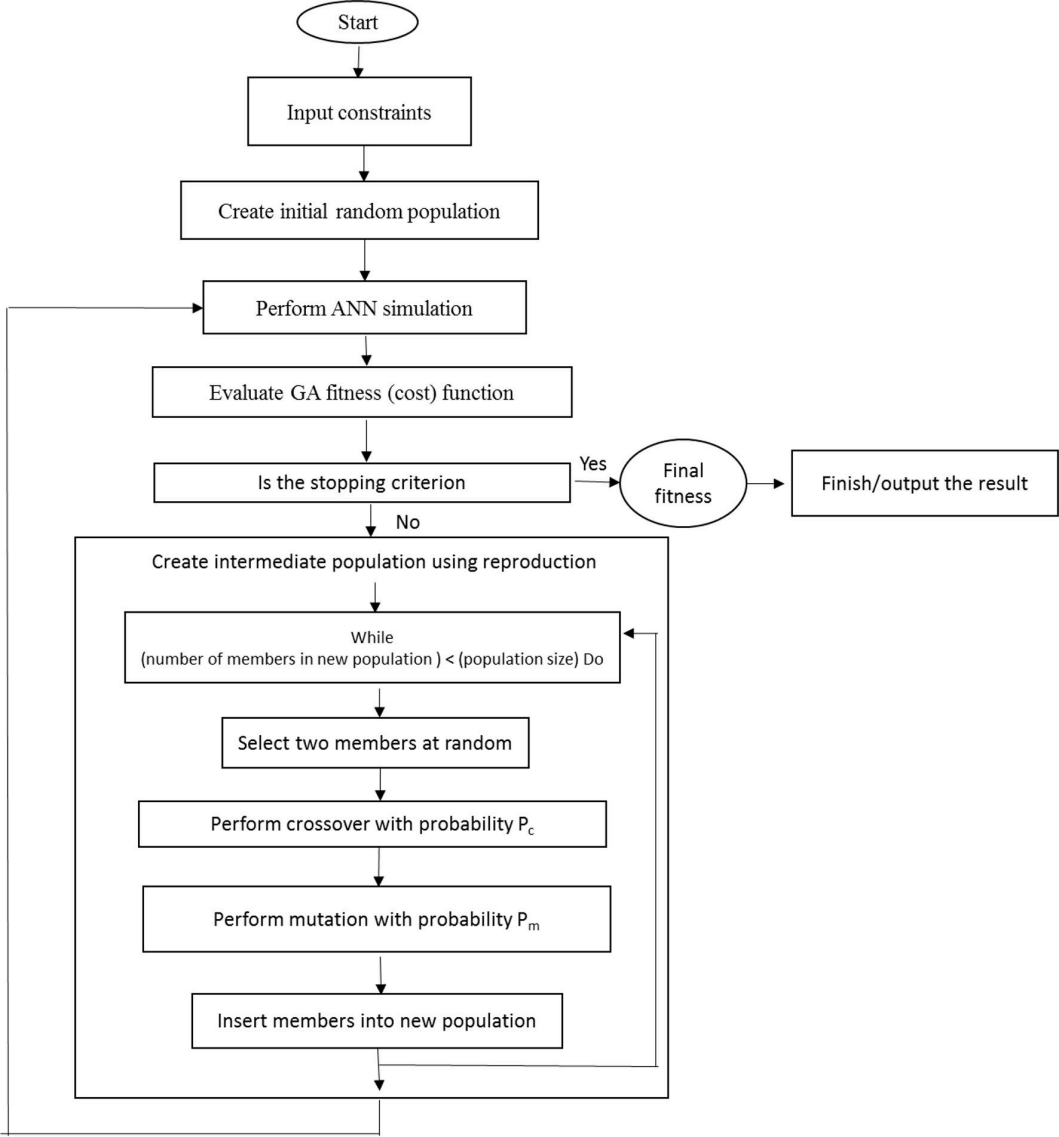
**Schematic describing the relationship between ANN and GA**.

The genetic algorithm (GA) is an optimization technique based on the biological principles of genetic variation and natural selection. It evolves finding the best solution for a specific problem. So, our main research objective was to analyze the ANN-GA models to address the question of how to get maximum PR, SL, and minimum STN, Chl, and Vitri (Figure 1).

Besides, ANN-GA can also be used in relation to other techniques such as stepwise regression modeling to achieve accurate results. To evaluate an appropriate model for nutrient medium both stepwise regression analysis and ANN-GA models were studied on a comparative base.

## Materials and methods

### Plant material and *in vitro* culture conditions

The experiments were carried out using micro-shoots of Pyrodwarf (“Old Home” × “Gute Luise”) and OHF (“Old Home” × “Farmingdel”) rootstocks from *in vitro* cultures. Briefly, micro-shoots were proliferated in MS (Murashige and Skoog, 1962) medium containing 2.5 mg/l BAP (6-benzylaminopurine), 0.2 mg/l IBA (indole-3-butric acid), 30 g/l sucrose, and 8 g/l agar (DuchefaH). Media pH was set to 5.7 and was placed into in 250- ml glass bottles with autoclave-resistant plastic caps (5.5 cm in diameter, 8 cm in height) prior autoclaving (121°C, 1 kg cm^−2^ s^−1^ for 15 min). The cultures were maintained under a 16 h-photoperiod at 80 μmol m^−2^ s^−1^ with a 16- h photoperiod of fluorescent bulbs light and at temperatures of 25 ± 2°C. For pyrodwarf rootstock, each treatment in each set of experiments consisted of 15 replicates and for OHF rootstock it consisted of 10 replicates. Each replication consisted of five jam jars of four explants each.

### Stepwise regression procedure

Regression analysis is one of the most common procedures for predictive modeling. A multiple regression model with more than one explanatory variable may be written as y = b_0_ + b_1_x_1_ + b_2_x_2_ +…+ b_p_x_p_, where y is the output variable, b_i_ the regression parameters (i = 0,1,2,…,p), x_i_ the input variables (i = 1,2,…,p). When regression coefficients are obtained, a prediction equation can then be used to predict the value of a continuous output as a linear function of one or more independent inputs. The popularity of the regression models may be attributed to the interpretability of model parameters and ease of use. In our application to the prediction of culture medium macro-nutrients composition, stepwise regression models are used, with both the entry and stay points for the models set to 0.05.

The stepwise regression analysis was carried out for the data obtained to test significance of the independent variables of KNO_3_, NH_4_NO_3_, CaCl_2_, MgSO_4_, and KH_2_PO_4_ affecting proliferation rate (PR; number of new regenerated micro-shoots), shoot length (SL; length of new regenerated micro-shoots in cm), shoot tip necrosis (STN) percentage, chlorosis (Chl) percentage, and vitrification (Vitri) percentage of pear rootstock explants as dependent variables.

### Optimization of explant growth parameters via Taguchi method

Taguchi design is a powerful and efficient tool for optimizing process that functions consistently and optimally over various conditions. Taguchi designs (orthogonal arrays) allow analyzing many factors with few runs. In such a design, no factor is weighted more or less in an experiment and thus allowing factors to be analyzed independently over each other. There are some noise factors which cause deviation of the functional characteristics of a product from their target values (e.g., human errors).

According to Taguchi's OA, a standard orthogonal array L_27_ (3^5^) 27 experiments with 26° of freedom were used to evaluate the effect of five factors (KNO_3_, NH_4_NO_3_, CaCl_2_, MgSO_4_, and KH_2_PO_4_) on SL, PR, Vitri, Chl, and STN. To all experiments, the three levels of factor variations were 1.25, 1.75, and 2.25 × MS medium concentrations and the designing of the L_27_ orthogonal array of Taguchi experimental design was illustrated as in Tables 1, 2. Each mineral concentration treatment consisted of at least 15 replicates of three explants each.

**Table 1:** **Ion concentrations of the various media types used for OHF and pyrodwarf rootstocks micropropagation and mean values of the parameters used to characterize it**.

**Rootstock**	**Culture medium**	**Ion Composition (mM)**								**PR**	**SL (cm)**	**STN**	**Chlo**	**vitri**
		**NO${}_{3}^{-}$**	**NH${}_{4}^{-}$**	**Ca^2+^**	**K^+^**	**Mg^2+^**	**PO${}_{4}^{2-}$**	**SO${}_{4}^{2-}$**	**Cl^−^**					
**OHF**	1	49.27	25.78	3.74	25.06	1.87	1.56	2.11	3.74	3.64	2.81	0	0	0
	2	49.27	25.78	3.74	25.67	1.87	2.18	2.11	3.74	3.25	2.97	1.25	0	0
	3	49.27	25.78	3.74	26.3	1.87	2.81	2.11	3.74	3.21	2.99	1.25	0	0
	4	59.58	36.09	5.24	25.06	2.63	1.56	2.87	5.24	2.74	3.38	2.38	0.75	0
	5	59.58	36.09	5.24	25.67	2.63	2.18	2.87	5.24	2.43	3.57	3.63	1.50	0
	6	59.58	36.09	5.24	26.3	2.63	2.81	2.87	5.24	2.06	4.03	1.75	0.50	0
	7	69.9	46.41	6.74	25.06	3.38	1.56	3.62	6.74	1.60	4.55	0.50	0.88	0
	8	69.9	46.41	6.74	25.67	3.38	2.18	3.62	6.74	2.60	3.47	1.00	0	0
	9	69.9	46.41	6.74	26.3	3.38	2.81	3.62	6.74	2.33	3.69	1.63	2.63	0
	10	58.66	25.78	5.24	34.44	3.38	1.56	3.62	5.24	3.79	2.47	5.75	1.13	0
	11	58.66	25.78	5.24	35.06	3.38	2.18	3.62	5.24	5.62	1.51	7.13	1.13	0
	12	58.66	25.78	5.24	35.69	3.38	2.81	3.62	5.24	7.38	1.07	8.88	1.00	0
	13	68.97	36.09	6.74	34.44	1.87	1.56	2.11	6.74	3.74	2.64	10.00	7.75	5.63
	14	68.97	36.09	6.74	35.06	1.87	2.18	2.11	6.74	3.51	2.84	14.00	11.13	6.13
	15	68.97	36.09	6.74	35.69	1.87	2.81	2.11	6.74	2.42	3.67	20.25	9.70	9.50
	16	79.29	46.41	3.74	34.44	2.63	1.56	2.87	3.74	2.23	3.78	20.88	5.63	11.50
	17	79.29	46.41	3.74	35.06	2.63	2.18	2.87	3.74	2.85	3.17	7.88	7.63	15.38
	18	79.29	46.41	3.74	35.69	2.63	2.81	2.87	3.74	3.76	2.53	7.25	7.13	19.38
	19	68.06	25.78	6.74	43.84	2.63	1.56	2.87	6.74	3.26	2.96	7.25	9.00	7.75
	20	68.06	25.78	6.74	44.46	2.63	2.18	2.87	6.74	2.67	3.46	4.63	10.38	1.00
	21	68.06	25.78	6.74	45.09	2.63	2.81	2.87	6.74	3.12	3.08	11.00	12.00	1.38
	22	78.37	36.09	3.74	43.84	3.38	1.56	3.62	3.74	3.70	2.57	30.75	14.25	2.63
	23	78.37	36.09	3.74	44.46	3.38	2.18	3.62	3.74	3.47	2.71	40.75	13.88	5.75
	24	78.37	36.09	3.74	45.09	3.38	2.81	3.62	3.74	1.64	4.56	19.63	14.25	11.00
	25	88.69	46.41	5.24	43.84	1.87	1.56	2.11	5.24	1.34	5.03	50.50	16.13	4.38
	26	88.69	46.41	5.24	44.46	1.87	2.18	2.11	5.24	1.00	5.41	55.50	19.00	4.63
	27	88.69	46.41	5.24	45.09	1.87	2.81	2.11	5.24	1.08	5.31	68.13	23.00	11.00
	MS	39.41	20.62	2.99	20.04	1.5	1.25	1.74	2.99	3.49	2.76	13.75	13.50	3.63
	QL	27.88	5	2.54	19.79	0.71	1.98	0.95	0	3.46	2.73	3.00	7.00	12.50
	WPM	9.71	5	3.01	6.93	1.5	1.25	7.42	0.65	2.58	3.52	6.13	12.13	5.50
**PYRODWARF**														
	1	49.27	25.78	3.74	25.06	1.87	1.56	2.11	3.74	11.23	3.43	0.17	0	0
	2	49.27	25.78	3.74	25.67	1.87	2.18	2.11	3.74	11.21	3.31	0.50	0	0
	3	49.27	25.78	3.74	26.3	1.87	2.81	2.11	3.74	10.91	3.38	0.50	0	0
	4	59.58	36.09	5.24	25.06	2.63	1.56	2.87	5.24	9.73	3.74	15.33	0.83	1.00
	5	59.58	36.09	5.24	25.67	2.63	2.18	2.87	5.24	9.47	4.13	18.67	1.50	0.17
	6	59.58	36.09	5.24	26.3	2.63	2.81	2.87	5.24	8.21	4.35	18.67	0.00	1.00
	7	69.9	46.41	6.74	25.06	3.38	1.56	3.62	6.74	6.99	4.83	20.17	2.33	5.33
	8	69.9	46.41	6.74	25.67	3.38	2.18	3.62	6.74	6.64	5.26	20.67	0.83	5.83
	9	69.9	46.41	6.74	26.3	3.38	2.81	3.62	6.74	6.02	5.09	21.83	0.00	8.67
	10	58.66	25.78	5.24	34.44	3.38	1.56	3.62	5.24	4.59	3.17	11.50	2.50	3.33
	11	58.66	25.78	5.24	35.06	3.38	2.18	3.62	5.24	4.73	3.27	11.83	10.50	1.17
	12	58.66	25.78	5.24	35.69	3.38	2.81	3.62	5.24	4.62	2.99	12.67	0	3.67
	13	68.97	36.09	6.74	34.44	1.87	1.56	2.11	6.74	4.92	3.49	27.33	0	3.83
	14	68.97	36.09	6.74	35.06	1.87	2.18	2.11	6.74	4.49	2.75	26.50	7.50	6.17
	15	68.97	36.09	6.74	35.69	1.87	2.81	2.11	6.74	4.53	3.39	26.17	3.50	6.33
	16	79.29	46.41	3.74	34.44	2.63	1.56	2.87	3.74	3.65	3.63	9.67	1.50	10.83
	17	79.29	46.41	3.74	35.06	2.63	2.18	2.87	3.74	3.47	4.12	10.50	19.33	13.33
	18	79.29	46.41	3.74	35.69	2.63	2.81	2.87	3.74	3.73	5.07	12.33	12.50	13.50
	19	68.06	25.78	6.74	43.84	2.63	1.56	2.87	6.74	6.31	4.90	25.00	6.33	4.50
	20	68.06	25.78	6.74	44.46	2.63	2.18	2.87	6.74	7.16	3.47	25.57	0.83	5.83
	21	68.06	25.78	6.74	45.09	2.63	2.81	2.87	6.74	6.05	3.47	26.33	3.17	3.50
	22	78.37	36.09	3.74	43.84	3.38	1.56	3.62	3.74	3.93	3.08	12.00	0.83	8.50
	23	78.37	36.09	3.74	44.46	3.38	2.18	3.62	3.74	3.88	3.07	13.17	3.67	10.17
	24	78.37	36.09	3.74	45.09	3.38	2.81	3.62	3.74	3.56	4.34	15.83	0.50	11.50
	25	88.69	46.41	5.24	43.84	1.87	1.56	2.11	5.24	2.31	4.01	20.67	1.17	21.67
	26	88.69	46.41	5.24	44.46	1.87	2.18	2.11	5.24	1.52	2.49	22.50	7.00	26.00
	27	88.69	46.41	5.24	45.09	1.87	2.81	2.11	5.24	1.05	3.29	22.17	12.00	30.00
	MS	39.41	20.62	2.99	20.04	1.5	1.25	1.74	2.99	3.77	3.29	0.50	8.67	7.33
	QL	27.88	5	2.54	19.79	0.71	1.98	0.95	0	3.06	2.47	12.50	7.33	0.50
	WPM	9.71	5	3.01	6.93	1.5	1.25	7.42	0.65	2.23	2.01	2.33	5.83	0.50

*Only ions with different concentration among media have been included*.

**Table 2:** **Levels of variables according to Taguchi—L_27_ orthogonal array**.

**Run**	Levels of factors used in each trial^*^				
	**KNO_3_**	**NH_4_NO_3_**	**CaCl_2_**	**MgSO_4_**	**KH_2_PO_4_**
1	1.25	1.25	1.25	1.25	1.25
2	1.25	1.25	1.25	1.25	1.75
3	1.25	1.25	1.25	1.25	2.25
4	1.25	1.75	1.75	1.75	1.25
5	1.25	1.75	1.75	1.75	1.75
6	1.25	1.75	1.75	1.75	2.25
7	1.25	2.25	2.25	2.25	1.25
8	1.25	2.25	2.25	2.25	1.75
9	1.25	2.25	2.25	2.25	2.25
10	1.75	1.25	1.75	2.25	1.25
11	1.75	1.25	1.75	2.25	1.75
12	1.75	1.25	1.75	2.25	2.25
13	1.75	1.75	2.25	1.25	1.25
14	1.75	1.75	2.25	1.25	1.75
15	1.75	1.75	2.25	1.25	2.25
16	1.75	2.25	1.25	1.75	1.25
17	1.75	2.25	1.25	1.75	1.75
18	1.75	2.25	1.25	1.75	2.25
19	2.25	1.25	2.25	1.75	1.25
20	2.25	1.25	2.25	1.75	1.75
21	2.25	1.25	2.25	1.75	2.25
22	2.25	1.75	1.25	2.25	1.25
23	2.25	1.75	1.25	2.25	1.75
24	2.25	1.75	1.25	2.25	2.25
25	2.25	2.25	1.75	1.25	1.25
26	2.25	2.25	1.75	1.25	1.75
27	2.25	2.25	1.75	1.25	2.25

* *The numbers are coefficients to product to the amounts in Table 1*.

### Artificial neural network model development, evaluation, and optimization

Here, we used one of the most famous network algorithms, i.e., the feed forward back-propagation (three-layer back-propagation network) consisted of input, output, and hidden layers and considered in making the ANN model (Demuth et al., 2006). The transfer functions used for the hidden and output layers were hyperbolic tangent sigmoid (tansig) and linear (purelin) functions, respectively. For training the network, a Levenberg-Marquardt algorithm for back-propagation with a gradient descent and momentum weight and bias learning function was used (Demuth et al., 2006). Used performance function was MS error with 0.01 level and training was terminated after 800 epochs or iterations of the network. Four input variables of KNO_3_, NH_4_NO_3_, CaCl_2_, MgSO_4_, and KH_2_PO_4_ with different levels were used as units in the input layer of the ANN model. Five models were developed separately for SL, PR and Vitri, Chl, and STN. 600 and 450 data lines were used to train and test the network. Before training, the data set (input and output data) was normalized in the range of −1 to 1 so as to make simpler the problem for the network, to attain fast conjunction minimum mean square error, and to make sure that the fall of targets (output data) into the particular range of the new feed forward network can be recreated (Demuth et al., 2006; Gulati et al., 2010; Ahmadi and Golian, 2011).

The fitness of the ANN-model was evaluated using R^2^, MSerror, and MBE (Ahmadi et al., 2007). In order to determine the optimal values of input variables (KNO_3_, NH_4_NO_3_, CaCl_2_, MgSO_4_, and KH_2_PO_4_requirements) and maximize SL and PR and minimize Vitri, Chl, and STN, the raised ANN models were exposed to a further process using GA, after the training practice. Thus, the ANN models were used as the fitness function for GA. A roulette wheel selection method was used for selecting elite populations for crossover. Initial population of 50, generation number of 500, mutation rate of 0.1, and crossover rate of 0.85 has been set to achieve the best fitness (Haupt and Haupt, 1998; The MathWorks., 2009). The generational practice was frequently performed to reach the number of generations. Through performing GA, the search for the optimal solutions was restricted between the input variable limits determined in the Taguchi design (Table 2). To recognize which input variable is more important in the model, the constructed ANN models were subjected to the process of the sensitivity analysis. This analysis shows which KNO_3_, NH_4_NO_3_, CaCl_2_, MgSO_4_, and KH_2_PO_4_ concentration is more important than the other to reach optimal SL, PR, Vitri, Chl, and STN of pear rootstock explants.

The sensitivity of SL, PR, Vitri, Chl, and STN against the investigating media nutrients was determined using the criteria (Lou and Nakai, 2001; Ahmadi and Golian, 2010a,b) as follows:

- The variable sensitivity error (VSE) value: which indicates the performance of the developed ANN model if that variable is unavailable,
- The value of variable sensitivity ratio (VSR): indicates the relative ratio between the VSE and the error of ANN model when all variables are available. A more important variable has a higher VSR value. Thus, based on the obtained VSR value, the input variables may be ranked in the order of importance.

Matlab R2010a (Matlab., 2010) software was used to write mathematical code to develop and evaluate the ANN-GA model. In point of fact, the developed program is a modified source code of an ANN algorithm which was formerly used by Ahmadi and Golian (2011).

## Results and discussion

The growth of *in vitro* plant tissues can be controlled by altering the culture media nutrients. Optimization of the media mineral contents is very laborious and time-consuming, and therefore predicting the favorable composition of the growth media and the culture conditions is very useful in order to achieve maximum productivity.

### Stepwise regression analysis

The stepwise selection method described here was used to evaluate the contributions of five minerals of culture media in the growth of *in vitro* pear rootstocks explants. These data were not previously analyzed by researchers in this area.

#### OHF rootstock

The stepwise regression model results are given in Table 3 (number of observations = 600). NH${}_{4}^{+}$, Mg^2+^, and SO${}_{4}^{2-}$ are important macro-nutrients influencing the Proliferation and NO${}_{3}^{-}$, NH${}_{4}^{+}$, Ca^2+^, K^+^, Mg^2+^, PO${}_{4}^{2-}$, and SO${}_{4}^{-}$are important macro-nutrients for obtaining optimum SL. On the other hand, NO${}_{3}^{-}$, K^+^, Mg^2+^, SO${}_{4}^{2-}$, and Cl^−^ are important factors affecting STN, NO${}_{3}^{-}$, NH${}_{4}^{+}$, Ca^2+^, K^+^, Mg^2+^, PO${}_{4}^{2-}$, SO${}_{4}^{2-}$, and Cl^−^ are important macro-nutrients influencing Chl and NO${}_{3}^{-}$, Mg^2+^, PO${}_{4}^{2-}$, SO${}_{4}^{2-}$, and Cl^−^ are important for explant Vitri phenomenon. As it is evident, Cl^−^ is the only macro-nutrient that just plays role in features which decrease the quality of the obtained plantlet. So, Cl^−^ plays a critical role in the culture medium and its appropriate amount should be further searched.

**Table 3:** **Stepwise regression model of optimized nutrient media for different measured *in vitro* factors of OHF pear rootstock**.

**Measured factor**	**Variable^a^**	**Parameter estimation**	**Standard error**
**PROLIFERATION**			
	Intercept^1^	3.55	0.19
	NH${}_{4}^{+}$	–0.08	0.00
	Mg^2+^	0.99	0.07
	SO${}_{4}^{2-}$	–0.16	0.05
*R*^2^		0.39	
RMSE		1.02	
MBE		0.13	
**SL**			
	Intercept	1.16	0.26
	NO${}_{3}^{-}$	0.35	0.05
	NH${}_{4}^{+}$	–0.28	0.05
	Ca^2+^	0.07	0.03
	K^+^	–0.33	0.05
	Mg^2+^	–0.90	0.06
	PO${}_{4}^{2-}$	0.35	0.08
	SO${}_{4}^{2-}$	0.35	0.05
*R*^2^		0.44	
RMSE		0.79	
MBE		–0.04	
**STN**			
	Intercept	–24.18	2.35
	NO${}_{3}^{-}$	0.78	0.05
	K^+^	0.71	0.08
	Mg^2+^	–14.53	0.86
	SO${}_{4}^{2-}$	5.18	0.52
	Cl^−^	–3.01	0.31
*R*^2^		0.67	
RMSE		10.22	
MBE		0.05	
**CHL**			
	Intercept	–78.05	14.97
	NO${}_{3}^{-}$	313.32	64.45
	NH${}_{4}^{+}$	–313.17	64.46
	Ca^2+^	–622.62	128.76
	K^+^	–312.71	64.46
	Mg^2+^	–318.56	64.46
	PO${}_{4}^{2-}$	312.10	64.38
	SO${}_{4}^{2-}$	314.43	64.44
	Cl^−^	621.96	128.75
*R*^2^		0.72	
RMSE		3.53	
MBE		0.39	
**VITRI**			
	Intercept	–4.64	0.97
	NO${}_{3}^{-}$	0.27	0.01
	Mg^2+^	–4.85	0.31
	PO${}_{4}^{2-}$	1.43	0.30
	SO${}_{4}^{2-}$	2.83	0.19
	Cl^−^	–1.50	0.11
*R*^2^		0.54	
RMSE		3.67	
MBE		–0.15	

a *Significant variable entered in the model. All variables left in the model are significant (p < 0.05)*.

#### Pyrodwarf rootstock

The stepwise regression model results are given in Table 4 (number of observations = 450). NH${}_{4}^{+}$, Ca^2+^, K^+^, PO${}_{4}^{2-}$, and Cl^−^, are found to be important factors influencing proliferation and NO${}_{3}^{-}$, NH${}_{4}^{+}$, Mg^2+^, and Cl^−^ are important for SL. NO${}_{3}^{-}$, NH${}_{4}^{+}$, Ca^2+^, K^+^, Mg^2+^, PO${}_{4}^{2-}$, and Cl^−^; NO${}_{3}^{-}$, Mg^2+^, SO${}_{4}^{2-}$ and Cl^−^; and NO${}_{3}^{-}$, Mg^2+^, SO${}_{4}^{2-}$, and Cl^−^ are important in the occurrence of STN, Chl, and Vitri phenomenon, respectively. Now, SO${}_{4}^{2-}$ is the macro-nutrient that plays role in features of STN, Chl, and Vitri of plantlets. So here, SO${}_{4}^{2-}$ plays a critical role in the culture medium and its appropriate amount should be further searched.

**Table 4:** **Stepwise regression model of optimized nutrient media for different measured in vitro factors of Pyrodwarf pear rootstock**.

**Measured factor**	**Variable^a^**	**Parameter estimation**	**Standard error**
**PROLIFERATION**			
	Intercept	15.00	0.66
	NO${}_{3}^{-}$	0.40	0.12
	NH${}_{4}^{+}$	–0.52	0.12
	Ca^2+^	–4.36	0.28
	K^+^	–0.61	0.13
	PO${}_{4}^{2-}$	0.94	0.20
	Cl^−^	4.59	0.25
*R*^2^		0.56	
RMSE		1.88	
MBE		1.41	
**SL**			
	Intercept	2.08	0.13
	NO${}_{3}^{-}$	–0.03	0.00
	NH${}_{4}^{+}$	0.06	0.01
	Mg^2+^	0.37	0.05
	Cl^−^	0.10	0.02
*R*^2^		0.46	
RMSE		0.64	
MBE		0.49	
**STN**			
	Intercept	–29.69	1.10
	NO${}_{3}^{-}$	–2.50	0.19
	NH${}_{4}^{+}$	2.72	0.19
	Ca^2+^	14.20	0.43
	K^+^	2.86	0.20
	Mg^2+^	–0.87	0.23
	PO${}_{4}^{2-}$	–2.01	0.31
	Cl^−^	–9.05	0.40
*R*^2^		0.89	
RMSE		2.91	
MBE		2.38	
**CHL**			
	Intercept	2.60	1.17
	NO${}_{3}^{-}$	0.13	0.02
	Mg^2+^	–2.39	0.43
	SO${}_{4}^{2-}$	1.02	0.26
	Cl^−^	–0.84	0.16
*R*^2^		0.17	
RMSE		4.48	
MBE		3.48	
**VITRI**			
	Intercept	–12.83	0.96
	NO${}_{3}^{-}$	0.52	0.01
	Mg^2+^	–6.13	0.36
	SO${}_{4}^{2-}$	2.46	0.22
	Cl^−^	–1.20	0.13
*R*^2^		0.77	
RMSE		3.69	
MBE		2.88	

a *Significant variable entered in the model. All variables left in the model are significant (p < 0.05)*.

### Artificial neural network analysis

Neural network software recently has been successfully applied for finding the optimal plant culture conditions (Zielinska and Kepczynska, 2013). ANNs are progressively applied in elucidation and analysis of the data in plant tissue culture experiments. Gago et al. (2010) developed a neural model to analyze the effect of two variables of sucrose and light on the proliferation of kiwifruit micro-shoots (*Actinidia deliciosa*). Nezami Alanagh et al. (2014) suggested that the macronutrients content of the culture media cause differences in GF677 explant growth. So, we used the optimal architecture of the multi-layer perceptron neural network to model the effect of the ion concentrations on the growth parameters of *Pyrus* rootstocks.

The predicted values and the optimization of explant growth parameters by the ANN-model are shown in Table 5. The comparison of observed and predicted outputs describes the behavior of the ANN-model from investigating inputs. The results revealed good agreements between the observed and the predicted values of explant growth parameters for training and testing sets (Table 6). The calculated statistics on the ANN-models are in close agreement to the two subsets in prediction of each output (statistics for both training and testing sets in Table 5). A well-trained ANN-model has a balance statistics values for these two subsets. This may suggest that over fitting has not occurred during the training process (Ahmadi and Golian, 2010b). The main advantage of ANN-model is that it does not require a prior specification of suitable fitting function thus; it has a universal approximation ability to approximate almost all kinds of non-linear functions. This flexibility feature may help the modeler to make a model with almost highest possible prediction accuracy.

**Table 5:** **Optimization analysis on artificial neural network (ANN) model to reach maximum PR and SL and minimum STN, Chl, and Vitri in OHF and Pyrodwarf pear rootstocks**.

**Item**	**Input variable [Ion concentrations (mM)]**								**Predicted output variable at optimal point**
	**NO${}_{3}^{-}$**	**NH${}_{4}^{+}$**	**Ca${}_{2}^{+}$**	**K^+^**	**Mg^2+^**	**PO${}_{4}^{2-}$**	**SO${}_{4}^{2-}$**	**Cl^−^**	
**OHF**									
PR	62.5	5.7	2.7	31.5	3.3	2.6	5.6	3.5	7.3
SL	88.1	42.8	6.3	40	2.3	2.2	4.6	4.9	5.5
STN	13.3	12.3	5.5	22.8	2.4	1.6	1.3	2.5	0
Chl	54.4	16.8	5.3	12.3	2	2.1	3.3	1.8	0
Vitri	48.9	33.9	4.8	9.4	2.1	2.1	7.1	4.9	0
**PYRODWARF**									
PR	25.6	13.1	5.5	35.7	1.5	2.1	3.6	3	11.4
SL	86.5	37.5	4.2	25.9	3.2	2.7	1.2	5.2	5.04
STN	20.9	22	3.5	28.1	2.1	1.3	2.7	6.1	0.2
Chl	54.8	14.7	2.5	45.1	0.7	2.8	0.9	6.7	0
Vitri	24.5	6.6	6.1	18.8	2.3	1.8	4.1	1.2	0

**Table 6:** **Statistics and information on artificial neural network models for number of shoots, SL, STN, Chl, and quality index of OHF and pyrodwarf rootstock during *in vitro* proliferation (training vs. testing values)**.

**Rootstock**	**Statistics1**	**Number of shoots**		**SL**		**STN**		**Chl**		**Vitri**	
**OHF**		**Training**	**Testing**	**Training**	**Testing**	**Training**	**Testing**	**Training**	**Testing**	**Training**	**Testing**
	*R*^2^	0.95	0.94	0.83	0.85	0.94	0.97	0.93	0.94	0.96	0.97
	RMSE	0.32	0.29	0.43	0.42	4.54	3.08	1.75	1.63	1.05	0.99
	MBE	–0.0009	–0.025	–0.0002	0.007	0.0003	–0.075	0.00002	–0.182	–0.0002	–0.067
**PYRODWARF**											
	*R*^2^	0.97	0.95	0.87	0.85	0.96	0.96	0.91	0.92	0.96	0.96
	RMSE	0.51	0.67	0.32	0.34	1.82	1.72	1.39	1.48	1.46	1.50
	MBE	0.00007	–0.05	0.0004	0.006	0.0002	–0.203	–0.0003	0.11	–0.0002	0.27

### Sensitivity analysis of the models

To determine the relative importance of input variables, the entire 27 data lines (training and testing) utilized to calculate the overall VSR for each plant growth parameter. The obtained VSR for the model output variables with respect to eight input variable are shown in Tables 7, 8.

**Table 7:** **Importance of ion concentrations (mM) of the different culture media used for Pyrodwarf rootstock micro-propagation according to the sensitivity analysis on the developed neural network model to rank the importance of ion concentrations**.

**Element**	**VSR**^a^							
	**NO${}_{3}^{-}$**	**NH${}_{4}^{+}$**	**Ca${}_{2}^{+}$**	**K^+^**	**Mg^2+^**	**PO${}_{4}^{2-}$**	**SO${}_{4}^{2-}$**	**Cl^−^**
PR	20.7	12.6	23.7	6.5	7.1	1.8	3.2	18.8
Rank	2	4	1	6	5	8	7	3
SL	7.1	10.7	7.7	3.5	7.9	4	5.9	6
Rank	4	1	3	8	2	7	6	5
STN	9.1	8	8.5	5.6	5.3	1.9	2.9	5
Rank	1	3	2	4	5	8	7	6
Chl	91.2	317.6	50.1	90.9	99.2	71.8	20.4	180.2
Rank	4	1	7	5	3	6	8	2
Vitri	49.7	79.6	14.6	25.9	7.9	11.3	15.5	12.8
Rank	2	1	5	3	8	7	4	6

a *Relative indication of the ratio between the variable sensitivity error and the error of the model when all variables are available*.

**Table 8:** **Importance of ion concentrations (mM) of the different culture media used for OHF rootstock micropropagation according to the sensitivity analysis on the developed neural network model to rank the importance of ion concentrations**.

**Element**	**VSR^a^**							
	**NO${}_{3}^{-}$**	**NH${}_{4}^{+}$**	**Ca${}_{2}^{+}$**	**K^+^**	**Mg^2+^**	**PO${}_{4}^{2-}$**	**SO${}_{4}^{2-}$**	**Cl^−^**
PR	90.5	301.7	127.9	197.5	150.4	18.5	149.1	113
Rank	7	1	6	2	3	8	4	5
SL	64	64	28.4	32.5	17.8	2.7	10.2	14.6
Rank	1	1	3	2	4	7	6	5
STN	6.2	15.2	22.6	19.6	17.1	10.2	54.1	24.8
Rank	8	6	3	4	5	7	1	2
Chl	16.8	19.1	15.9	40.4	5.7	9.4	27	37.3
Rank	5	4	6	1	8	7	3	2
Vitri	35.1	30.3	22.3	12.1	11.6	8.1	1.9	3.9
Rank	1	2	3	4	5	6	8	7

a *Relative indication of the ratio between the variable sensitivity error and the error of the model when all variables are available*.

A more important input variable has a higher VSR value. Thus, the investigating inputs can be ranked according to their importance of effect on outputs using VSR values (Tables 7, 8). Among the input variables, NH${}_{4}^{+}$ (301.7) and NO${}_{3}^{-}$, NH${}_{4}^{+}$ (64), SO${}_{4}^{2-}$ (54.1), K^+^ (40.4), and NO${}_{3}^{-}$ (35.1) in OHF and Ca^2+^ (23.7), NH${}_{4}^{+}$ (10.7), NO${}_{3}^{-}$ (9.1), NH${}_{4}^{+}$ (317.6), and NH${}_{4}^{+}$ (79.6) in Pyrodwarf had the highest values of VSR in data set, respectively, for PR, SL, STN, Chl, and Vitri. According to the obtained VSR values, SL was more sensitive to NO${}_{3}^{-}$ (64) and NH${}_{4}^{+}$ (64) followed by K^+^ (32.5), Ca^2+^ (28.4), Mg^2+^ (17.8), Cl^−^ (14.6), SO${}_{4}^{2-}$ (10.2), and PO${}_{4}^{2-}$ (2.7) in OHF and NH${}_{4}^{+}$ (10.7) followed by Mg^2+^ (7.9), Ca^2+^ (7.7), NO${}_{3}^{-}$ (7.1), Cl^−^ (6), SO${}_{4}^{2-}$ (5.9), PO${}_{4}^{2-}$ (4), and K^+^ (3.5) in Pyrodwarf.

### Model optimization

The optimization analysis on the ANN model to maximize PR exhibited that media containing 62.5 mM NO${}_{3}^{-}$, 5.7 mM NH${}_{4}^{+}$, 2.7 mM Ca^2+^, 31.5 mM K^+^, 3.3 mM Mg^2+^, 2.6 mM PO${}_{4}^{2-}$, 5.6 mM SO${}_{4}^{2-}$, and 3.5 mM Cl could lead to optimal PR for OHF and optimal PR for Pyrodwarf may be obtained with media containing containing 25.6 mM NO${}_{3}^{-}$, 13.1 mM NH${}_{4}^{+}$, 5.5 mM Ca^2+^, 35.7 mM K^+^, 1.5/, mM Mg^2+^, 2.1 mM PO${}_{4}^{2-}$, 3.6 mM SO${}_{4}^{2-}$, and 3 mM Cl^−^. The optimal point for SL may be acquired with media containing 88.1 mM NO${}_{3}^{-}$, 42.8 mM NH${}_{4}^{+}$, 6.3 mM Ca^2+^, 40 mM K^+^, 2.3 mM Mg^2+^, 2.2 mM PO${}_{4}^{2-}$, 4.6 mM SO${}_{4}^{2-}$, and 4.9 mM Cl^−^ for OHF rootstock and 86.5 mM NO${}_{3}^{-}$, 37.5 mM NH${}_{4}^{+}$, 4.2 mM Ca^2+^, 25.9 mM K^+^, 3.2 mM Mg^2+^, 2.7 mM PO${}_{4}^{2-}$, 1.2 mM SO${}_{4}^{2-}$, and 5.2 mM Cl^−^ for Pyrodwarf rootstock. Results of our investigations showed that media including 13.2 mM NO${}_{3}^{-}$, 12.3 mM NH${}_{4}^{+}$, 5.5 mM Ca^2+^, 22.8 mM K^+^, 2.4 mM Mg^2+^, 1.6 mM PO${}_{4}^{2-}$, 1.3 mM SO${}_{4}^{2-}$, and 2.5 mM Cl^−^ required for minimum STN in OHF and 20.9 mM NO${}_{3}^{-}$, 22 mM NH${}_{4}^{+}$, 3.5 mM Ca^2+^, 28.1 mM K^+^, 2.1 mM Mg^2+^, 1.3 mM PO${}_{4}^{2-}$, 2.7 mM SO${}_{4}^{2-}$, and 6.1 mM Cl^−^ in Pyrodwarf. In OHF rootstock, media with 54.4 mM NO${}_{3}^{-}$, 16.8 mM NH${}_{4}^{+}$, 5.3 mM Ca^2+^, 12.3 mM K^+^, 2 mM Mg^2+^, 2.1 mM PO${}_{4}^{2-}$, 3.3 mM SO${}_{4}^{2-}$, and 1.8 mM Cl^−^ could lead to the optimal point for Chl, whereas in Pyrodwarf rootstock the optimal point for Chl could be achieved with media supplemented with 54.8 mM NO${}_{3}^{-}$, 14.7 mM NH${}_{4}^{+}$, 2.5 mM Ca^2+^, 45.1 mM K^+^, 0.7 mM Mg^2+^, 2.8 mM PO${}_{4}^{2-}$, 0.9 mM SO${}_{4}^{2-}$, and 6.7 mM Cl^−^. Finally, The optimization results of ANN-GA models revealed that media containing 48.9 mM NO${}_{3}^{-}$, 33.9 mM NH${}_{4}^{+}$, 4.8 mM Ca^2+^, 9.4 mM K^+^, 2.1 mM Mg^2+^, 2.1 mM PO${}_{4}^{2-}$, 7.1 mM SO${}_{4}^{2-}$, and 4.9 mM Cl^−^ in OHF and 24.5 mM NO${}_{3}^{-}$, 6.6 mM NH${}_{4}^{+}$, 6.1 mM Ca^2+^, 18.8 mM K^+^, 2.3 mM Mg^2+^, 1.8 mM PO${}_{4}^{2-}$, 4.1 mM SO${}_{4}^{2-}$, and 1.2 mM Cl^−^ in Pyrodwarf could lead to optimal Vitri. Gago et al. (2010) developed a neural model to analyze the effect of two variables: sucrose and light on the proliferation of kiwi fruit microshoots (*Actinidia deliciosa*). Nezami Alanagh et al. (2014) found that the macronutrients content seems to be responsible for the observed differences in GF677 explant growth. So we used the optimal architecture of the multi-layer perceptron neural network to model the effect of the ion concentrations on the growth parameters of *Pyrus* rootstocks that was suggested by “intelligent problem solver” was found with eight inputs, five outputs (with linear activation function), and 10 hidden neurons (with hyperbolic tangent activation function). A training algorithm of Quasi–Newton was used to train the network (Lou and Nakai, 2001). The optimization of the architecture by the intelligent problem solver was performed on the basis of the “balance error against diversity” option. This option tries to produce a structure with a balance performance value against type and diversity. It will preserve networks with a range of types and performance/ complexity trade-offs. This may led to obtain an optimized ANN-model with less complexity and more accuracy (Tahmoorespur and Ahmadi, 2012). The ANN-model was successfully used to describe associations between investigating 8 macronutrients and explant growth parameters. The sensitivity analysis on the ANN-model indicated that NH${}_{4}^{+}$ and NO${}_{3}^{-}$ in OHF and Ca^2+^ and NH${}_{4}^{+}$ in Pyrodwarf are the most important variables respectively in the PR and SL. SO${}_{4}^{2-}$, K^+^ and NO${}_{3}^{-}$ in OHF and NO${}_{3}^{-}$, NH${}_{4}^{+}$, and again NH${}_{4}^{+}$ in Pyrodwarf are the most important variables respectively in STN, Chl, and Vitri. Our results indicated that there are differences in explant responses to macronutrient concentrations in different pear rootstock genotypes. Improved explant growth in OHF required increased NH${}_{4}^{+}$ in combination with low SO${}_{4}^{2-}$ and K^+^. NO${}_{3}^{-}$ is critical for OHF since despite increasing its concentration can improve SL but it causes Vitri disorder if it increases more than a critical point (Table 5). High Ca^2+^ and low NO${}_{3}^{-}$ are required for improved explant growth in Pyrodwarf but now, NH${}_{4}^{+}$ concentration is critical which increasing can cause explant Chl and Vitri. So, it can be suggested that the use of ANN-base model analyses allows us to realize the best macronutrient concentrations required to maximize the explant growth parameters like PR and SL and minimize the explant physiological disorders like STN, Chl, and Vitri which were investigated in the present study.

### Comparison of ANN-GA and stepwise regression models

For output variables (PR, SL, STN, Chl, and Vitri) the calculated statistical values corresponding to the ANN-GA revealed a substantially higher accuracy of prediction than for regression models (as calculated R^2^ for ANN-GA vs. regression models were: PR = 0.94 vs. 0.39, SL = 0.85 vs. 0.44, STN = 0.97 vs. 0.67, Chl = 0.94 vs. 0.72, Vitri = 0.96 vs. 0.54 for OHF, and PR = 0.95 vs. 0.56, SL = 0.85 vs. 0.46, STN = 0.96 vs. 0.89, Chl = 0.92 vs. 0.17, Vitri = 0.96 vs. 0.77 for Pyrodwarf). According to the ANN-GA results, among the input variables, NH${}_{4}^{+}$ (301.7) and NO${}_{3}^{-}$, NH${}_{4}^{+}$ (64), SO${}_{4}^{2-}$ (54.1), K^+^ (40.4), and NO${}_{3}^{-}$ (35.1) in OHF and Ca^2+^ (23.7), NH${}_{4}^{+}$ (10.7), NO${}_{3}^{-}$ (9.1), NH${}_{4}^{+}$ (317.6), and NH${}_{4}^{+}$ (79.6) in Pyrodwarf have the highest values of VSR in data set, respectively, for PR, SL, STN, Chl, and Vitri. The ANN-GA showed that media containing 62.5 NO${}_{3}^{-}$, 5.7 NH${}_{4}^{+}$, 2.7 Ca, 31.5 K^+^, 3.3 Mg^2+^, 2.6 PO${}_{4}^{2-}$, 5.6 SO${}_{4}^{2-}$, and 3.5 Cl^−^ could lead to optimal PR for OHF and optimal PR for Pyrodwarf may be obtained with media containing containing 25.6 NO${}_{3}^{-}$, 13.1 NH${}_{4}^{+}$, 5.5 Ca^2+^, 35.7 K^+^, 1.5 Mg^2+^, 2.1 PO${}_{4}^{2-}$, 3.6 SO${}_{4}^{2-}$, and 3 Cl^−^.

In order to develop an optimized protocol, it is important to use a reliable modeling system to reach optimal growth and productivity. There is much statistical software for optimizing the growth medium for *in vitro* plant culture (Gago et al., 2010; Gallego et al., 2011). Response surface method (RSM) is one of the software that has been repeatedly used to optimize growth medium for *in vitro* culture of pear genotypes (Reed et al., 2013a,b; Wada et al., 2013, 2015). Previous studies detected that ANN-GA models had a substantially higher accuracy of prediction than RSM models (Sedghi et al., 2012). Moghri et al. (2015) indicated that RSM alone is not trustworthy for approximation of non-polynomial or non-linear variables.

Additionally, it has been shown that GA is easy, precise and efficient method (Moghri et al., 2015), which can be helpful for establishing an optimized culture medium. Neural models have been developed for modeling the effect of different culture conditions of plant tissue culture on explant growth such as sucrose and light (Gago et al., 2010) and macro-nutrients content (Nezami Alanagh et al., 2014). Several investigations have revealed that high concentrations of plant growth regulators (PGRs) cause somatic variations (Karp, 1992; Martin et al., 2006). Additionally, some authors have reported that PGRs (especially cytokinins) can cause STN in several woody plant species (Kataeva et al., 1991; Piagnani et al., 1996). The effectiveness of the media optimization for the reduction of essential PGRs was investigated in several studies (Preece, 1995).

Optimal nutrient media is required for appropriate explant growth. N is considered of the critical nutrients for explant growth which is mainly supplied as nitrate or ammonium in the culture media (Engelsberger and Schulze, 2012). The type and amount of supplied N may be genotype dependent. Clearly, nitrate is the suitable kind of supplied N for most plant species, and it is the accessible form of N in most *in vitro* media (Sathyanarayana and Blake, 1994; Ivanova and Van Staden, 2009; Nezami Alanagh et al., 2014). The optimization analysis on the ANN model showed that NO${}_{3}^{-}$ is important for OHF explant growth since regardless of increasing its concentration it can improve growth parameters (Reed et al., 2013a,b). This indicates that NO${}_{3}^{-}$ is needed in amounts higher than its content in MS medium for the best growth of OHF explant. Similarly, some investigations showed that high amounts of NO${}_{3}^{-}$ are required for improving shoot multiplication and length (Shirdel et al., 2011; Hand et al., 2014). Also, Bell et al. (2009) and Mamaghani et al. (2010) both reported that low N content in culture media caused some physiological disorders in several pears. On the contrary, low NO${}_{3}^{-}$ improves some growth factors in Pyrodwarf but pear genotypes react differentially in this regard (Wada et al., 2015). In both studied rootstocks, increase in NO${}_{3}^{-}$ more than a critical range will cause STN disorder (Tables 7, 8). This finding corresponds to those studies which suggested that reducing salts concentrations by changing culture medium from MS to WPM or half-strength MS (1/2MS), decreased STN (Grigoriadou et al., 2000; Bairu et al., 2009; Jain et al., 2009). Conversely, Reed et al. (2013a,b) noted that high amounts of N compounds improved STN in some pear species.

The sensitivity analysis on the ANN-GA model revealed that NH${}_{4}^{+}$ is a key nutrient in the propagation of pear genotypes. Results showed that shooting is sensitive to high NH${}_{4}^{+}$ concentrations. High NH${}_{4}^{+}$ concentrations significantly diminished the mean number of shoot per explant. This may be due to the significant inhibitory effects of NH${}_{4}^{+}$ on other ionś uptake (Gerendás et al., 1997; Lorenzo et al., 2000). Moreover, some investigations suggested that high amounts of NH${}_{4}^{+}$ adversely effect on plant metabolism causing to physiological and morphological disorders. Previously, negative effect of high NH${}_{4}^{+}$ on shoot elongation has been reported in few studies (Gamborg and Shyluk, 1970). Conversely, in our study, the high NH${}_{4}^{+}$ was not associated with shoot length reduction. It has been proved that high concentrations of NH${}_{4}^{+}$ induce Vitri in some plant species (Ivanova and Van Staden, 2008; Gago et al., 2011; Reed et al., 2013a,b). This may be due to the fact that high amount of NH${}_{4}^{+}$ induces ethylene production in the culture media (George, 1993). (Gaspar, 1991) concluded that increasing the ethylene owing to raising the concentration of NH${}_{4}^{+}$ could prompt a series of events leading to Vitri. It has been believed that the toxic effects of high NH${}_{4}^{+}$ concentrations increase the activity of glutamate dehydrogenase which in turn, cause a shift in carbohydrate pool from lignin synthesis to amino acid synthesis (Beauchesne, 1981), leading to Vitri (Letouzé and Daguin, 1983; Brand, 1993). The opposite response was found for Pyrodwarf as several investigations reported no Vitri on shoots grown on high levels of NH${}_{4}^{+}$ in culture media (Nezami Alanagh et al., 2014; Wada et al., 2015). Our results showed that high concentrations of NH${}_{4}^{+}$ and amounts of Cl^−^ and K^+^ in the culture medium were the most important factors influencing explant Chl of studied pear rootstocks. This conclusion is in agreement with Perez-Tornero et al. (2001) who found that Chl could be the result of high NH${}_{4}^{+}$ concentrations. From these results, it is confirmed that NH${}_{4}^{+}$ concentrations has a strong effect on micro-propagation of pear rootstocks.

Ca^2+^ is a relatively large essential cation for cation-anion balance in plant tissues (Martin et al., 2007). Shacklock et al. (1992) and Hepler (2005) both concluded that Ca^2+^ has a significant role on physiological and developmental processes. It is clearly evident that Ca^2+^ is a key element in the structure and physiological properties of cell membranes (Sha et al., 1985; Hirschi, 2004). Also, it plays an important role in the synthesis and activity of several crucial enzymes (George, 1993). Studies on some plants showed that Ca^2+^ is an essential factor for protocorm formation (Mitra et al., 1976), adventitious bud formation (Tanimoto and Harada, 1986), increase the mean number of somatic embryos (Jansen et al., 1990), somatic embryogenesis (Timmers et al., 1996), formation of meristemoids (Capitani and Altamura, 2004), and facilitated the uptake of some nutrients (Aranda-Peres et al., 2009). These facts show that why Ca^2+^ is critical for improving some disorders in plants (Singha et al., 1990) and is necessary for plant growth and development. In agreement with our findings, in several studies have been demonstrated that high Ca^2+^ concentrations ameliorate the STN disorder (Wang and van Staden, 2001; Chang and Miller, 2005; Martin et al., 2007; Bairu et al., 2009). Similar to the findings of those results, ANN-GA models revealed that high concentrations of Ca^2+^ are required for optimum growth and development of pear genotypes (Tables 7, 8). Nevertheless, this is not often true so that some studies have reported that high concentrations of Ca^2+^ increase the STN percentage in some pear genotypes (Grigoriadou et al., 2000; Thakur and Kanwara, 2011).

It is evident that ANN-GA models clearly illustrate the independent role of K^+^ in micro-propagation of pear rootstocks (Tables 7, 8). The results show that high concentrations of K^+^ is necessary for increasing shoot multiplication, shoot elongation and relieving the STN disorder in both studied rootstocks. It might be due to the important function of K^+^ in protein synthesis and maintenance of sufficient turgor for growth (Leigh and Wyn Jones, 1984). Our results are contradictory with Ramage and Williams (2002) who noted that low levels of K^+^ are required for plant growth and differentiation. Also, the present results showed that high K^+^ concentration lead to explant Vitri in both rootstocks. In opposition, Pasqualetto et al. (1988) showed that Vitri increased with low levels of K^+^.

## Conclusion

The ANN-model was successfully used to describe associations between investigating eight macro-nutrients and explant growth parameters. The sensitivity analysis on the ANN-model indicated that NH${}_{4}^{+}$ and NO${}_{3}^{-}$ in OHF and Ca^2+^ and NH${}_{4}^{+}$ in Pyrodwarf are the most important variables respectively in the PR and SL. SO${}_{4}^{2-}$, K^+^ and NO${}_{3}^{-}$ in OHF and NO${}_{3}^{-}$, NH${}_{4}^{+}$ and again NH${}_{4}^{+}$ in Pyrodwarf are the most important variables respectively in STN, Chl, and Vitri. Our results indicated that there are differences in explant responses to macronutrient concentrations in different pear rootstock genotypes. High Ca^2+^ and low NO${}_{3}^{-}$ are required for improved explant growth in Pyrodwarf but now, NH${}_{4}^{+}$ concentration is critical which increasing can cause explant Chl and Vitri. Improved explant growth in OHF required increased NH${}_{4}^{+}$ in combination with low SO${}_{4}^{2-}$ and K^+^. NO${}_{3}^{-}$ is critical for OHF since despite increasing its concentration can improve SL but it causes Vitri disorder if it increases more than a critical range. So, it can be suggested that the use of ANN-base model analyses allows us to realize the best macronutrient concentrations required to maximize the explant growth parameters like PR and SL and minimize the explant physiological disorders like STN, Chl, and Vitri which were investigated in the present study.

## Author contributions

All authors listed, have made substantial, direct and intellectual contribution to the work, and approved it for publication.

### Conflict of interest statement

The authors declare that the research was conducted in the absence of any commercial or financial relationships that could be construed as a potential conflict of interest.
